# A Multidimensional Perspective on Resilience in Later Life: A Systematic Literature Review of Protective Factors and Adaptive Processes in Ageing

**DOI:** 10.3390/geriatrics10060154

**Published:** 2025-11-21

**Authors:** Benjamin A. Jacob, Cameron Walker, Michael O’Sullivan, Paul Rouse, Matthew Parsons

**Affiliations:** 1 Department of Engineering Science and Biomedical Engineering, University of Auckland, Auckland 1010, New Zealand; cameron.walker@auckland.ac.nz (C.W.); michael.osullivan@auckland.ac.nz (M.O.); 2 Department of Accounting and Finance, University of Auckland, 12 Grafton Road, Auckland 1010, New Zealand; p.rouse@auckland.ac.nz; 3 Division of Health, University of Waikato, Gate 4-Hillcrest Road, Hamilton 3240, New Zealand; matthew.parsons@waikato.ac.nz

**Keywords:** resilience, aged, healthy aging, protective factors, risk factors, social support, self-efficacy

## Abstract

**Background:** With the global population rapidly aging, resilience has emerged as a critical determinant of healthy aging. While many factors are associated with resilience, a comprehensive synthesis is needed to inform targeted interventions and policy. **Objectives:** This systematic review aimed to identify and synthesize the conceptual models and key protective factors associated with resilience in older people. **Methods:** Following PRISMA guidelines, a systematic literature search was conducted in Web of Science, PubMed, PsycNet, and JSTOR for studies published between 2017 and 2025. Search terms included (including synonyms and closely related words) “resilience,” “older people,” and “models.” Studies were screened based on relevance to resilience models, measurement tools, and associated factors. Included studies underwent a formal risk of bias assessment. **Results:** From 7109 initial records, 54 studies met the inclusion criteria. Ten studies explored conceptual models, while 44 investigated contributing factors. Resilience was predominantly assessed using standardized psychometric tools. Findings were synthesized by mapping key determinants across Macro-Environmental, Meso-Social, Micro-Individual and Bio-Physiological domains. **Conclusions:** Resilience in later life is a dynamic and multifactorial process, not a fixed trait. The evidence suggests a range of modifiable factors at various levels that can be targeted to support wellbeing. An integrated, systems-based perspective is essential for guiding future research and developing effective interventions to promote resilience across the aging trajectory.

## 1. Introduction

The World Health Organization (WHO) states that Healthy ageing concerns the development and maintenance of functional ability, which is the capability of a person to do what they have reason to value [1]. All countries are encountering rapid growth in the number and proportion of older people [2]. World Population Prospects 2022 stated that the number of people above the age of 65 is growing more rapidly than the number of people below that age [3]. According to the United Nations’ population projections, the Global share of older people aged 65 and above increased from 5.5 percent to 10.3 percent between 1974 and 2024 [4]. They predict that this number will double again between 2024 and 2074. At the same time, the number of people aged 80 and above is expected to triple. Even though developed countries have the highest number of older people, developing countries (e.g., China, India, South Africa) are also witnessing rapid population ageing [4,5,6,7]. It is expected that the number of people above the age of 65 will be twice the number of children under the age of five and almost equal to the number of children under twelve by 2050. In other words, older people are expected to outnumber youths globally. According to the WHO, by 2030, one in six people worldwide will be aged 60 years and above [8].

As people age, the accumulation of a wide variety of molecular and cellular damage also increases over time, leading to a gradual decline in physical and cognitive capacity. Some common issues that occur in older age are sensory loss, osteoarthritis, depression, and dementia. Despite these issues, a longer life brings many opportunities as well [9,10]. Older people can contribute in many ways to their families and communities, but most importantly, their contributions depend on one factor: health. If one can experience good health in the later years of life, the ability to do things of value will be much higher. The concept of healthy ageing arose from two observations: the possibility of differences in how individuals experience ageing and the continually increasing human lifespan [11,12]. ‘Individual aging’ refers to the phenomenon that although people may share the same chronological age, they often differ significantly in their biological age, functional ability, and health status [12]. In other words, the rate at which people experience physical, cognitive, and emotional changes over time varies significantly from person to person. People’s longevity and health depend on successful dynamic interactions between biological, psychological, social, and environmental factors [13].

Resilience, or the ability to adapt well and recover effectively in the face of adversity, is one of the key components of successful aging [14]. Resilience encompasses psychological, emotional, and behavioral processes that enable individuals to maintain their wellbeing and effectively cope with the demands of ageing [15]. Research has shown a strong association between resilience and healthy ageing. It has been found that people who are highly resilient experience greater joy and life satisfaction, which in turn leads to healthier ageing [16]. Thus, it is critical that the basis of resilience be better understood and measures to assess it be developed. This enables the development of treatments and preventive strategies to improve physical resilience and identify older adults who are most vulnerable during medical and surgical procedures. Resilience can be viewed from two perspectives: the classical perspective sees it as a positive response to high-intensity stressors, while the newer perspective focuses on how individuals maintain or return to equilibrium over time in response to frequent, low-intensity stressors [17]. By nurturing resilience in people, they can be well-equipped to overcome and cope with the adversities associated with ageing. Hence, resilience is important in maintaining the wellbeing of older people, who face health-related and social adversities. Resnick suggests that resilience facilitates personal growth among older individuals as they adapt and overcome various life challenges [18]. It has also been reported that conducting resilience enhancement programs focusing on older people helps alleviate the adverse effects of high-intensity stressors [19,20]. Therefore, even though ageing is associated with reductions in performance across multiple areas, resilience acts as a protective shield for older people, helping them to preserve functional abilities and achieve wellbeing. Research in the field of ageing increasingly emphasizes the pivotal role of resilience in maintaining wellbeing and promoting healthy ageing among older people [21,22,23,24]. Thus, within ageing research, resilience is an emerging and valuable concept, the exploration of which could identify opportunities for improving the wellbeing of older people [25]. Identifying resiliency models to determine protective factors may increase understanding of resilience from both individual and public health perspectives [26].

Drawing from other disciplines provides a broader understanding of resilience. In fields like engineering and finance, resilience is conceptualized as a system’s ability to both withstand a shock and the speed at which it recovers to a stable, functional state afterwards [21,27]. Applying this to gerontology, resilience in older people can be understood not just as the capacity to endure a stressor, such as an illness or fall, but also the efficiency and completeness of their functional recovery.

Previous research and studies on ageing have focused on the factors that contribute to resilience and resilience scales [28,29]. However, a comprehensive review of implementing resilience strategies in older people has re-emphasized the importance of a conceptual model for resilience as a first step towards implementing resilience strategies [30]. As interest in the concept of resilience among older individuals is growing, a comprehensive overview of the literature on resilience models and factors affecting resilience is warranted [17].

This review was conducted to identify and summarize the foundational conceptual models used to frame resilience in later life, and to systematically identify and synthesize the empirical evidence on the key protective and risk factors associated with resilience in older adults. Furthermore, a key aim of this review is to integrate these identified factors into a comprehensive, multilevel perspective [31]. To achieve this, we mapped the determinants of resilience across Macro-Environmental, Meso-Social, Micro-Individual and Bio-Physiological domains. This will enable the development of treatments and preventive strategies to enhance physical resilience and promote a healthy lifestyle. Thus, this literature survey presents a mix of cross-sectional and longitudinal studies.

## 2. Research Methods

The protocol for this systematic review was registered on the Open Science Framework (OSF) (https://doi.org/10.17605/OSF.IO/JC482). This systematic review was conducted and reported in accordance with the Preferred Reporting Items for Systematic Reviews and Meta-Analyses (PRISMA) 2020 statement.

### 2.1. Literature Search

This section presents a systematic literature review on resilience in the elderly population. The search was conducted in two parts. The primary search was a systematic review of the literature on protective and risk factors for resilience in older adults. To gather relevant literature, a structured search strategy was employed across multiple electronic databases, covering publications from 2017 to 2025. This specific period was selected strategically to update and add to the findings of a comprehensive systematic review covering the literature on this issue from 1990 to 2018 [17]. By beginning our search in 2017, we created a brief overlap to capture any relevant articles that might have been published during the final stages of the former review’s production. Such a methodology was consequently deliberately chosen to avoid redundant analyses and focus on the most recent conceptual developments. The literature search and screening were performed between January 2025 and May 2025. The articles were collected from Web of Science, PubMed, PsycNet, and Journal Storage (JSTOR). The whole search string for the Web of Science database is provided below as an example:

((((((((((((((((((TI = (physical resilience)) OR TI = (financial resilience)) OR TI = (health resilience)) OR TI = (social resilience)) OR TI = (psychological resilience)) OR TI = (economic resilience)) OR TI = (spiritual resilience)) OR TI = (cultural resilience)) OR TI = (community resilience))) AND TI = (older adults)) OR TI = (aging)) OR TI = (elderly)) OR TI = (aged))))))

Publication Date: 1 January 2017 to 30 May 2025

Document Types: Article or Review Article or Proceeding Paper or Early Access or Book Chapters

Languages: English

This search string was adapted to meet the specific syntax requirements of the other databases. The full search strings for all databases are available in the Appendix (Appendix A).

In parallel, a targeted non-systematic secondary search was conducted to identify foundational or seminal conceptual models of resilience. This search was not restricted by publication date, allowing for the inclusion of key theoretical frameworks that provide the essential context for the recent empirical literature. The search was informed by expert consultation with the broader research team (CW, MO, MP, PR). Studies identified in this secondary search were included to frame the review, but the main synthesis focused on the empirical articles identified within our systematic search timeframe. The search string was broad, encompassing direct terms such as “resilience model,” “resilience framework,” “conceptual model of resilience,” and “theory of resilience in aging.”

There are some limitations to the search strategy. One limitation is that the search was confined to a specific set of databases (e.g., PubMed, PsychNet, JSTOR) and may have failed to capture articles that were exclusively indexed in other relevant databases. Another limitation is that our search strategy involved combinations of selected keywords and index terms; it is possible that articles that used different terminology for the concepts of resilience and ageing could have been missed.

### 2.2. Screening and Inclusion Criteria

All the articles were effectively managed using the EndNote tool. Any duplicates were first removed using EndNote’s Duplicate Finder, followed by three levels of screening: title screening, Abstract Screening, and Full-Text Screening. The inclusion criteria for title screening were the presence of the terms (including synonyms and closely related words) resilience, older adults, and models. In the first two rounds, articles were evaluated by a single reviewer (BJ), as these were straightforward to judge. The specific inclusion and exclusion criteria applied during this process are detailed in Table 1.

In full-text screening, the articles were thoroughly assessed. At this stage, a primary reviewer (BJ) thoroughly assessed all full-text articles against the pre-specified inclusion and exclusion criteria. To minimize selection bias and ensure methodological rigor, a second independent reviewer (CW) verified all articles excluded by the primary reviewer. Discrepancies were discussed to reach a consensus. In case of continuing disagreement or uncertainty, it was discussed with a broader research team (MO, MP, PR) to decide on inclusion/exclusion.

Methodological quality and risk of bias were assessed for each included study using appropriate JBI Critical Appraisal Checklists [32]. Thus, checklists for Cohort Studies, Cross-Sectional Studies, and Quasi-Experimental Studies were used, depending on the article’s design.

**Figure 1:**
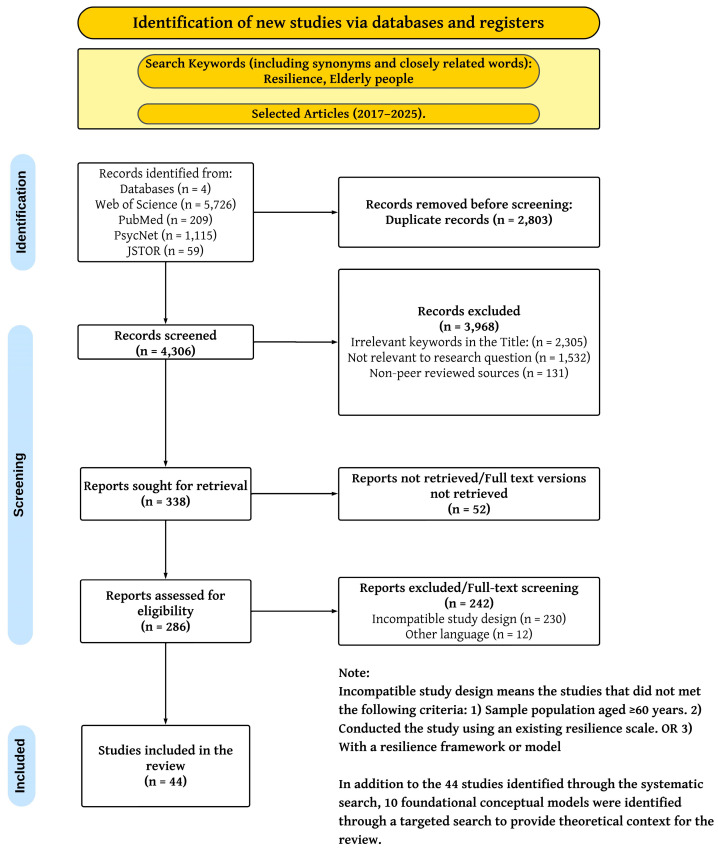
Flowchart Depicting Study Identification, Screening, and Inclusion (PRISMA).

To ensure reliability, a dual-reviewer process was implemented. An initial 10% sample of the studies was independently assessed by two reviewers (BJ and CW). The results were then compared, and discrepancies were resolved through consensus-based discussion. This practice calibrated the use of the appraisal tool. Afterwards, the primary author (BJ) conducted the quality appraisal of the remaining 44 studies. A detailed summary of the quality appraisal results for each included study is provided in Supplementary File, Table S1. The process flow for the systematic search (first 44 studies on protective and risk factors for resilience) is outlined in the flow diagram, as per PRISMA guidelines, in Figure 1.

## 3. Results

### 3.1. Included Studies

The systematic search yielded a total of 7109 studies. These were mostly found from the Web of Science database. After duplicate removal, title screening, and abstract screening, 286 full-text articles were assessed for eligibility. A final selection of 44 empirical studies met the inclusion criteria for the systematic review. In addition to these, 10 foundational conceptual models were identified through a targeted, non-systematic search to provide theoretical context. This resulted in a total of 54 studies being included in the final synthesis.

### 3.2. Characteristics of Studies on Key Resilience Factors

The number of participants in the selected studies ranged from 22 to 12,823. The mean age ranged from 64.5 to 88, and the age range of the entire cohort spanned from 60 to 99. All studies were published between 2017 and 2025. Most studies employed a cross-sectional study design and measured resilience using one of the following: the Connor-Davidson Resilience Scale (CD-RISC) [33], the Brief Resilience Scale (BRS) [34], or the Wagnild Resilience Scale (RS) [35].

The most popular Resilience Measurement Method is the Connor-Davidson Resilience Scale (CD-RISC), which has been used in 13 out of the 44 studies. It was primarily applied to samples of community-dwelling older people [36,37,38,39,40,41,42,43,44,45]. One study used the scale with hospitalized patients [46,47], and another with adults in a rural municipality [48]. For the studies that reported reliability within their sample, the internal consistency was consistently high, with Cronbach’s alpha (α) values ranging from 0.84 to 0.968.

The BRS was used in five studies, the majority of which also focused on community-dwelling older people [49,50,51,52]. One study applied the BRS to a sample of hospitalized older people [53]. The reported Cronbach’s alpha values for the BRS were also high, ranging from 0.81 to 0.90.

The RS was used in eight studies. While primarily used with community-dwelling older people [24,54,55,56,57,58,59], one study notably applied it to a sample of hospitalized rehabilitation patients [60]. Only one of the included studies reported a reliability score for the RS in its sample, which was 0.85.

These are psychometric resilience scales, which are standardized instruments used to measure an individual’s ability to adapt, bounce back, or recover from stress, adversity, or significant life challenges. A detailed summary of the 44 studies, which focus on the factors contributing to resilience in older people, is presented in Table 2. In addition to psychometric resilience scales, other tools used to measure resilience included clinical biomarkers, physiological measures, and functional health and recovery measures. The latter category encompasses assessments such as Activities of Daily Living (ADLs) and Instrumental Activities of Daily Living (IADLs).

The methodological characteristics of the 44 included studies reveal that the most popular Study Design-Resilience Measurement Method pair is the Cross-Sectional study with the Connor-Davidson Resilience Scale (CD-RISC). It also shows that Cross-sectional studies are most popular, followed by Longitudinal cohort studies.

### 3.3. Synthesis of Key Resilience Factors

The thematic synthesis of the 44 included studies revealed a multidimensional set of correlates of resilience in older people. These were categorized into protective factors (positively associated with resilience) and risk factors (negatively associated with resilience). The study-specific details are provided in Table 2.

#### 3.3.1. Protective Factors

Protective factors were the most extensively reported correlates of resilience, spanning psychological, social, physical, cognitive, and spiritual domains. Psychological and intrapersonal factors were the most consistently identified in 10 studies. The emphasis was on the strong positive association between resilience and higher self-efficacy, mastery, perceived control, and purpose in life [53,55,61,62,63]. Additional studies emphasized the importance of self-esteem [44,55], life satisfaction [39,64,65,66], and other constructs such as mindfulness and self-compassion [63,66].

Following closely, social and interpersonal factors were emphasized in 13 studies. Factors such as social engagement and participation [17,37,39,41,45,47,51,52,54,57,58,67,68] and family support [17] were consistently associated with higher resilience. Marital status was also reported as a protective factor in three studies [44,69,70].

The review also found strong evidence for the role of physical health and functional ability, with 18 studies reporting a positive association between resilience and indicators like functional status, ADL independence, physical activity, and mobility [17,36,37,45,46,49,51,53,59,60,66,67,68,69,70,71,72,73]. Health-promoting behaviors such as good nutrition [59] were also found to be protective. Furthermore, higher cognitive ability and executive functioning were identified as protective in at least 13 studies [37,45,46,49,59,66,67,68,69,73,74,75,76], while spiritual wellbeing was highlighted in two studies [40,43] as a source of meaning and a buffer against adversity. Genetics was also found to be a protective factor in one study [58].

Finally, while not a commonly reported theme, protective factors such as age, gender, education, quality of life, Pre-illness Functioning income, sleep quality, happiness, and secure attachment style were also identified in the review [24,42,44,45,63,68,69,70,74].

#### 3.3.2. Risk Factors

Although fewer in number, risk factors showed strong and consistent associations across studies.

The review also identified several key risk factors consistently associated with lower resilience. The most significant of these was psychological distress, with depression being the most frequently reported risk factor across at least nine [40,45,51,53,60,62,69,70,75,76]. Anxiety and stress were also reported as being negatively associated with resilience in multiple studies [45,50,64,75,76]. Furthermore, physical frailty, disability, and chronic illness, including conditions like hypertension, diabetes, and mobility limitations, were identified as significant risk factors [24,68,70]. Finally, lifestyle and health behaviors such as smoking and poor sleep quality were also found to be negatively associated with resilience [70].

Together, these findings suggest that resilience in older people is shaped by an interplay of intrapersonal resources, social connections, health status, and lifestyle factors, with psychological distress emerging as the most consistent risk factor.

### 3.4. Risk of Bias of Included Studies

The results of the quality appraisal, along with a detailed breakdown, are presented in Supplementary File, Table S1. Subsequently, studies were classified as good, moderate, or poor according to their alignment with the research objectives. No studies were excluded due to poor quality; however, most studies were identified as having a moderate quality. Of the studies, 10 (23%) were rated as having a low risk of bias (High quality), and 34 (77%) were rated as having a moderate risk of bias (Moderate quality).

**Table 2:** Summary of studies examining factors contributing to resilience in older people.

Study	Population Characteristics	Resilience Measurement Method	Key Protective Factors	Key Risk Factors
Taylor A.M. et al., 2019 [49]	sample size = 655, age ~ 73–79, mean age = 76	BRS	Cognitive ability, physical fitness, and wellbeing	-
Akkila S. et al., 2023 [71]	sample size = 79, age = 70–88, mean age = 75	EORTCQLQ-C30	Functional ability	-
Choi J.Y., 2024 [61]	Sample size = 57, age ≥ 65	ACTH stimulation test, orthostatic blood pressure measurement, dual-task gait tests	Personal competence (Self-efficacy, purposefulness)	-
Costenoble A. et al., 2022 [36]	Sample size = 405, age = 80–97, mean age = 83	CD-RISC	Activities of Daily Living (ADLs)	-
Costenoble A. et al., 2023 [37]	Sample size = 322, mean age 83.04	CD-RISC	ADLs, social participation, and psychological resilience	-
Drazich B.F. et al., 2025 [72]	Sample size = 314, mean age = 82.74	Physical Resilience Scale	Physical resilience	-
Gijzel S.M.W. et al., 2017 [67]	Sample size = 22, age ≥ 70, mean age = 84.0	Self-Rated Health Monitoring	Physical, Mental, and Social Health	-
Gijzel S.M.W. et al., 2020 [64]	Sample size = 121, mean age = 84.3	Dynamic Resilience Indicator	Health and life satisfaction.	Anxiety
Hao M. et al., 2024 [68]	Sample size = 1754, age = 70–84	Physiological System Network model	Age, Gender, education, cognition, Functional status, physical, mental, and social health	Disease history
Hu F.W. et al., 2021 [69]	Sample size = 192, age = 65–97, mean age = 76.29	PRIFOR	Gender, marital status, education, health, mental health, ADLs, quality of life	Depression, frailty
Hu F.W. et al., 2024 [73]	Sample size = 413, age ≥ 65, mean age = 76.34	PRIFOR)	Physical and mental health	-
Jyväkorpi S.K. et al., 2018 [59]	Sample size = 394, age = 82–97, mean age = 88	Finnish version of Resilience scale	Good nutrition, physical activity, and psychological health	-
Kim E. et al., 2024 [50]	Sample size = 1826, age = 70–84, mean age = 77.6	BRS	-	Stress
Kolk D. et al., 2022 [74]	Sample size = 207, ag ≥ 70, mean age = 79.8	Single Baseline Measurements scales	Pre-illness Functioning, Cognitive function	-
Lenti M.V. et al., 2022 [46]	Sample size = 143, median age = 69	CD-RISC	Functional ability, cognitive ability, and education	-
Miller M.J. et al., 2024 [62]	Sample size = 3778, mean age = 75.4	Modified Poisson regression analyses	Perceived control over one’s health	Depression
Kim S. et al., 2024 [75]	Sample size = 1397, age = 72–90, mean age = 82.2	Parallel-serial mediation model using PROCESS macro	Cognitive function	Depression and stress
Olson K. et al., 2021 [51]	Sample size = 3199, mean age = 72.2	BRS	Social support, physical activity	Depression
Rebagliati G.A. et al., 2017 [60]	Sample size = 81, age = 60–94	RS	Functional status	Depression
Rodrigues F. and Tavares D., 2024 [48]	Sample size = 201, age ≥ 60	CD-RISC- 25BRASIL	ADLs, self-perceived health	History and depression
Rolandi E. et al., 2024 [76]	Sample size = 404, age = 83–87	Multidimensional Assessment	Executive functions (skills like planning, problem-solving, and mental flexibility), lifestyle	Depression and anxiety
Stenroth S.M. et al., 2023 [77]	Sample size = 681, age = 71–84	Hardy-Gill resilience scale	-	Frailty
Sugawara I. et al., 2022 [54]	Sample size = 1064, age = 74–86	RS-14	Social engagement	-
Taylor M.G. and Carr D., 2021 [55]	Sample size = 11,050 to 12,823	SRS	Mastery (control over one’s life) and high self-esteem	-
Yang Y. and Wen M., 2017 [24]	Sample size = 11,112, age = 65–84, mean age = 82.9	Resilience Scale	Age	Disability
Ye B. et al., 2024 [38]	Sample size = 4033, mean age = 71.0	CD-RISC	-	Frailty
Wang Y. et al., 2024 [47]	Sample size= 280, age= 60–95, mean age= 74.21	CD-RISC	Social support	-
Jiang G.-q. et al., 2024 [70]	Sample size = 2495, age ≥ 60	SRQS	Marital status, monthly income, sleep quality, ADLs, and physical exercise	Smoking, depression, and hypertension
Wister A.V. et al., 2021 [39]	Sample size = 13,064, age ≥ 65, mean age = 73.75	CD-RISC	Social support, life satisfaction	-
Zafari M. et al., 2023 [40]	Sample size = 384, age ≥ 60, mean age = 67.41	CD-RISC	Spiritual wellbeing	Depression
Angevaare M.J. et al., 2020 [17]	Sample size = 246, age ≥ 60	interRAI-LTCF	Cognitive function, family support, social engagement, health, mental health	-
Remm S.E. et al., 2023 [53]	Sample size = 143, age ≥ 65, mean age= 79	BRS	Self-efficacy, mobility difficulties, and physical activity	Depression
Kim J.R. et al., 2023 [41]	Sample size = 284, age ≥ 60, mean age= 68	CD-RISC	Social participation	-
Li Y.T. et al., 2022 [65]	Sample size = 226, age ≥ 60	RSOA	Life satisfaction	-
Silva R.C.M. et al., 2022 [42]	Sample size = 65, mean age ≥ 65, men age = 71.32	CD-RISC	Happiness	-
Kunuroglu F. et al., 2021 [66]	Sample size = 264, age = 60–96, mean age = 70.29	ARM	Physical and Psychological health, satisfaction, and engagement with life, self-compassion	-
Asch R.H. et al., 2021 [63]	Sample size = 3001, age = 60–99, mean age = 73.2	PRAPDI	Secure attachment style, mindfulness, and purpose in life	-
Bartholomaeus J.D. et al., 2019 [52]	Sample size = 110, mean age = 70.69	BRS	Social participation	-
Lau S. et al., 2018 [56]	Sample size = 1506, age = 65–74, mean age = 69.5	RS	Health	-
Morete M.C. et al., 2018 [43]	Sample size = 108, mean age = 79.9	CD-RISC	Health, spiritual wellbeing	-
Kondabi F. et al., 2017 [44]	Sample size = 500, age = 60–69, mean age = 64.5	CD-RISC	Self-esteem, marital status, income, age	-
Phillips S.P. et al., 2017 [57]	Sample size = 1724, age = 65–74, mean age = 69.5	RS	Social engagement	-
Laird K.T. et al., 2019 [45]	Sample size = 337, age = 60–89, mean age = 70.45	CD-RISC	Quality of Life, Mental Health, Vitality, Apathy, Social functioning, Emotional Wellbeing, General Health, Age	Depression, Anxiety
Resnick B. et al., 2019 [58]	Sample = 172, age = 65–96, mean age = 81.09	RS	Genetics and Social Interaction	-

### 3.5. Resilience Models

Old age is often characterized by increasing disability, a growing number of diseases, and the disproportionate use of healthcare services. Hence, it is important to analyze the resilience factors that empower older people to locate these within a well-defined structure. Regarding resilience models, several models have been identified that consolidate a set of resilience factors. An ecological model based on individual stress and coping processes was proposed, in which resilience is defined as a process that unfolds over time within a particular context and is not merely a characteristic of a person alone. It is the process of coping with stress that leads to post-traumatic and stress-related growth. Resilience is the transactional relationship between multiple levels of resources. The most common levels of resources are individual, contextual, and sociocultural [78].

It is also possible to study resilience from a deeper level, such as the cellular or biological level and the physiological level. The society-to-cells model of resilience suggests that every person is born with some resilient potential and that potential changes over time based on the interaction between society, community, family, individual, physiological, and cellular factors. Thus, resilience is a lifelong process, not a fixed trait, which is impacted by continuous top-down and bottom-up influences across levels. When individuals experience a stressor, their patterns of response can vary significantly. Some individuals can resist the stressor altogether, maintaining their original level of physical and mental functioning without any decline. Others experience a temporary or lasting decrease in functioning. Among these, some individuals may not fully recover, remaining at a lower level of functioning compared to their pre-stressor state. However, others demonstrate resilience by gradually recovering to their original level of functioning. In some cases, individuals recover and achieve a higher level of functioning than before, reflecting growth and positive adaptation in response to adversity. These different trajectories highlight the diverse ways resilience can be manifested in response to challenges [31].

Resilience in older people is largely influenced by the resources available to them. In this context, resources refer to the internal and external factors that empower individuals to adapt positively to challenges and maintain or regain their wellbeing. Among these influences, individual resources or factors play a key role in building the resilient characteristics in an older adult. The main individual factors identified in the review are education, health, financial status, spirituality, self-efficacy, and sense of purpose. Table 3 describes all the models identified in the study.

## 4. Discussion

The literature overwhelmingly endorses the conceptualization of resilience in later life as a complex and dynamic phenomenon, influenced by the interplay between psychological, social, individual, family, and biological domains [90,91]. Resilience cannot be attributed to a single protective factor but rather is brought about through the dynamic interactions across these domains. For instance, it has been found that social support may buffer the negative outcomes associated with a decline in physical health, thereby demonstrating the interdependent nature of resilience mechanisms’ characteristics [92,93]. Additionally, the establishment and maintenance of resilience are shaped to a great extent by various contextual and temporal forces, such as a person’s life experiences, socioeconomic status, and cultural context [94,95]. These variables are a substantial cause of the varying resilience paths observed in older individuals and bear testimony to the importance of applying a detailed and descriptive approach when researching resilience in older adults.

Compared to the conceptual focus of the earlier review by Angevaare et al. [17], our synthesis of recent empirical literature confirms the multi-level nature of resilience, while providing robust evidence for specific protective and risk factors. Our findings reveal a notable increase in studies examining determinants across Macro-Environmental, Meso-Social, Micro-Individual and Bio-Physiological domains, and utilizing quantitative psychometric scales. This approach addresses a potential limitation noted by Angevaare et al. [17] regarding an overemphasis on individual aspects. This suggests the research pathway is productively advancing from conceptual exploration towards empirical measurement and testing of resilience factors in older adults.

### 4.1. Methodological Considerations in Resilience Measurement

A key finding of this systematic review was the consistent use of three primary psychometric scales to operationalize resilience: the CD-RISC, BRS, and RS. As a deeper comparative analysis of these instruments is warranted, a detailed breakdown of their theoretical underpinnings, target populations, and psychometric properties is presented in Table 4.

Our synthesis of the included literature reveals that the application of these well-established scales was heavily concentrated on community-dwelling populations. The reported reliability within these samples, as measured by Cronbach’s alpha, was consistently high, which strengthens confidence in the validity of these tools for non-clinical, community-based research.

Furthermore, the original validation studies provide robust evidence for the validity of these scales, demonstrating strong convergent and concurrent validity through expected correlations with related constructs (e.g., hardiness, optimism, life satisfaction) and health outcomes (e.g., stress, depression), alongside good discriminant validity and evidence supporting their theoretical factor structures [33,34,35].

However, this concentration also highlights a significant gap in the current evidence base: the limited application and validation of these scales within hospitalized or other clinical settings. For instance, our review found only a few studies that employed these tools in an inpatient context. This suggests that while the existing instruments are robust for studies of the general older population, their validity and reliability for more frail, clinical populations may be less established. Therefore, a critical direction for future research is to validate these resilience measures specifically within inpatient and long-term care settings to ensure they are appropriate for the full spectrum of the aging population.

### 4.2. Macro-Environmental Level Factors

At the Macro-Environmental level, broad societal and structural determinants play a significant role in influencing the possibility of resilience among older people. However, while interpersonal and socioeconomic factors were well-represented, our systematic search yielded limited evidence on the direct impact of broader structural determinants. We found a significant gap in the literature concerning factors such as a safe and accessible physical environment, the quality of health and social services, and specific social policies. Secure housing, neighborhoods that can be walked in, and age-friendly built environments act as a protection against the negative impacts of ageing-related decline [1]. Meanwhile, the presence and quality of health and social services are also critical to facilitating resilience. Easy access to healthcare, community services, and mental health services improves older adults’ capacity to cope with chronic illness, recover from health crises, and stay in their own homes [39]. In addition, social policies emphasizing income security, healthcare fairness, housing stability, and the safeguarding of elders directly affect the resources that older individuals have [96]. At the policy level, recommendations include investing in “age-friendly city” initiatives to foster social engagement, integrating geriatric mental health services into primary care to address depression, a key risk factor. At the community level, organizations should implement targeted programs, such as skill-building workshops, to enhance mastery and self-efficacy.

Additionally, they should offer social opportunities, like mentorship or walking groups, to foster support and purpose, as well as accessible physical activity classes to improve health. In this manner, resilience is not solely an individual or interpersonal process but is located in more extensive social structures and policies that facilitate or limit adaptive capacity.

### 4.3. Meso-Social Level Factors

The Meso-Social level encompasses the immediate social contexts, including both community and family domains, that significantly foster resilience. Our review consistently highlighted the importance of these close interpersonal relationships and community-level resources. The most prominent factor was social support, which was identified as a key contributor in multiple studies [39,47,51,68]. Social support networks within communities—e.g., senior centers, intergenerational programs, and volunteer groups—emotionally and instrumentally aid the individual, and both are reliably associated with more positive adaptation outcomes [97]. Similarly, active social engagement and participation in community activities were found to be highly protective [17,37,41,45,52,54,57,58,66,68].

Within the family domain, close interpersonal relationships and a protective environment serve as a critical foundation for resilience in later life. Our review confirmed that interpersonal relationships are a critical resource. Family support was explicitly identified as a key factor [17], and marital status was found to be associated with resilience in several analyses [44,69,70]. Older people with good relationships with spouses, children, or close relatives tend to experience greater life satisfaction, self-esteem, and optimism, despite declining health or loss [98]. Looking into the family’s protective function, it goes beyond caregiving, involving advocacy, decision-making, and preserving cultural or spiritual identity [98]. Accordingly, the family is not merely a source of support but a dynamic system working to promote and build resilience in ageing.

Furthermore, an increasing body of academic research is exploring the role of spiritual and cultural factors, particularly in non-Western and indigenous contexts. These factors, rich in meaning and identity, serve as sources of continuity that help individuals cope with adversity. However, their potential is still not fully realized, highlighting the need for more culturally responsive theories within resilience studies [99,100].

### 4.4. Micro-Individual Level Factors

Micro-Individual level factors, for instance, build a critical foundation for the development and maintenance of resilience in later life through their direct influence on behavioral, cognitive, emotional, and physiological adaptation mechanisms. At the individual level, our review identified a broad and critical foundation of factors that directly influence resilience. The most frequently examined determinants were related to an individual’s physical health and functional capacity. Multiple studies confirmed the importance of maintaining physical fitness, functional ability, and the capacity to perform Activities of Daily Living (ADLs), with some specifically conceptualizing this as a form of physical resilience [37,46,49,51,53,59,68,69,70,71,72]. Physically active individuals tend to enjoy better health outcomes, and physical activity also has a neurobiological effect, aiding in the regulation of stress and emotion, and promoting emotional resilience. In this respect, physical activity helps release endorphins and brain-derived neurotrophic factor (BDNF), which stabilize mood and strengthen cognitive function while lessening inflammatory responses—the very building blocks of resilience [101].

Alongside physical health, a set of core psychological traits consistently emerged as powerful intrapersonal resources. These included a strong sense of self-efficacy, mastery or control over one’s life, high self-esteem, and a clear sense of purposefulness [39,44,53,55,61,63,65,102]. To view self-efficacy and self-esteem as crucial intrapersonal resources, one might assess their importance in determining how older people appraise situations of adversity and respond to them. According to Bandura’s [103] social cognitive theory, those with high self-efficacy believe they have the capacity to attain control over an outcome; thus, they activate more adaptive coping strategies and remain motivated during aforementioned setbacks. These traits have been associated with reduced vulnerability to depression and proactive health behaviors, both of which are key to resilient ageing.

Furthermore, spiritual wellbeing was identified as an important existential resource for navigating adversity [40]. Although sometimes sidelined in Western-centric models of resilience, spirituality offers existential frameworks that facilitate older people in making sense of loss, ageing, and uncertainty. A spiritual worldview may serve as a meaning-making system that calms or emotionally stabilizes individuals who belong to culturally and religiously diverse groups [104].

While not a commonly reported theme, financial status was also identified as a protective factor in the review [70]. Having financial resources may provide access to healthcare and environments that are compatible with autonomy and agency-promoting factors, which predict wellbeing outcomes for the construct of resilience, such as self-rated health and quality of life [105].

Finally, key demographic and socioeconomic characteristics, such as Age [24,44,45,68], gender [68], and income [44,70], were also found to be significant individual-level factors. Gender differences appear relevant, as research indicates that socialization patterns and coping styles may advantage women in late life in terms of resilience, especially in relational or spiritual spheres [57]. Together, these findings illustrate that individual resilience is a multidimensional construct built upon a person’s physical, psychological, and socioeconomic assets.

### 4.5. Bio-Physiological Level Factors

Notably, our review of the literature from 2017 to 2025 revealed a significant research gap. Of the 44 studies included, the vast majority focused on psychological, social, and behavioral factors. A few studies touched upon general health status [17,36,37,40,43,45,46,49,50,53,56,59,62,66,68,69,70,73,74,76], but except one [58] none directly measured mechanisms like DNA inheritance, mitochondrial functioning, oxidative stress, cellular senescence and epigenetic regulation and physiological factors such as chronic inflammation in relation to resilience [106,107,108,109,110,111]. This suggests that, although the theoretical basis for these connections is strong, empirical research on the biological underpinnings of resilience in older adults remains a relatively new field. Our review, therefore, highlights a critical need for future studies to bridge this gap between the psychosocial and biological dimensions of resilience in aging.

A key area of interest in modern gerontology is the role of resilience in the context of multimorbidity. While not a dominant theme, our systematic review identified two studies that specifically examined resilience factors in older adults managing multiple chronic conditions [45,58]. The findings from these studies align with the broader conclusions of a recent scoping review by Seong et al. [112], which highlighted the complexity of managing treatment burdens and adapting to functional limitations in this population. Although our review captured some evidence in this domain, the relatively small number of studies meeting our criteria suggests that resilience in the context of multimorbidity remains a crucial and underdeveloped area for future research, particularly for developing interventions tailored to this high-needs group.

The results obtained from this systematic review should be interpreted with consideration of several limitations.

The search was limited only to studies in the English language. This may create a language bias, as other relevant research studies on resilience in older people may be published in languages other than English, thereby limiting the worldwide applicability of our results.

Secondly, there is a risk of publication bias in our review. We did not include a search of “grey literature,” which consists of unpublished dissertations, conference proceedings, and other similar materials. It is a fact that studies with statistically significant or positive results are more likely to get published than those with null or negative results. It is therefore possible that our findings represent a more positive association with resilience.

Lastly, there is considerable heterogeneity across included studies in terms of study design, population characteristics, and the specific instruments to measure resilience. While this heterogeneous nature provides a broad understanding of the topic, it did not allow for direct comparison of findings between studies and ruled out the conduct of a quantitative meta-analysis. Our synthesis is therefore narrative and provides a broad thematic overview rather than a precise statistical summary.

## 5. Conclusions

This systematic review synthesized the evidence on conceptual models and the protective and risk factors influencing resilience in later life. Resilience is best understood as a multidimensional and dynamic process that emerges from the interplay between Macro-Environmental, Meso-Social, Micro-Individual and Bio-Physiological domains, as underscored by various ecological and systems-based frameworks.

Our analysis of 44 empirical studies identified consistent protective factors, including individual resources (e.g., self-efficacy, purpose), socio-community resources (e.g., social support, engagement), and biological factors (e.g., physical function, cognitive capacity). Conversely, the most significant risk factors were psychological distress (primarily depressive symptoms), chronic disease, and frailty.

Mapping these factors onto the four levels such as Macro-Environmental, Meso-Social, Micro-Individual and Bio-Physiological, provides robust empirical support for a multilevel perspective, demonstrating that resilience arises from a dynamic interconnection of resources and challenges across different levels. The validity and reliability of our research findings are underscored by this integrated view, which has clear implications: interventions must be multifaceted, targeting both individual capacities and supportive contextual factors to be effective.

In conclusion, fostering resilience offers a clear, evidence-based pathway to promoting healthy and meaningful aging. Future research must prioritize longitudinal studies to understand resilience trajectories, investigate the under-explored biological mechanisms and the specific context of multimorbidity, and rigorously evaluate multilevel interventions designed to enhance the capacity of older adults to adapt and thrive amidst challenges.

## Figures and Tables

**Table 1:** Inclusion and Exclusion Criteria for the Systematic Review.

Criteria Domain	Inclusion Details	Exclusion Details
Population	Studies focused on adults aged 60 and above, or with a mean sample age of 60 or older.	Studies focused exclusively on younger or middle-aged adult populations.
Concept/Content	For empirical studies: Must investigate protective/risk factors associated with resilience AND quantitatively measure resilience using a validated or explicitly defined scale. For theoretical papers: Must primarily explore or present a conceptual model of resilience relevant to aging.	Studies where resilience was mentioned only in passing, without being a primary focus of the research.
Publication Type	Peer-reviewed journal articles (quantitative, qualitative, or mixed-methods). Book chapters or review articles presenting foundational conceptual models.	Dissertations, conference abstracts, editorials, book reviews, and other non-peer-reviewed “grey literature.”
Publication Date	For empirical studies on factors: Published between January 2017 and October 2025. For foundational conceptual models: No date restriction.	For empirical studies on factors: Published before 2017.
Language	Full-text article published in English.	Articles published in languages other than English.
Accessibility	Full text retrievable through university library subscriptions or inter-library loan.	Full text not accessible.

**Table 3:** Resilience models and factors are identified in each model.

Model & Author(s) (Year)	Core Concept/Definition	Key Components/Levels	Key Contribution to Understanding Resilience in Aging
An Ecological Model of Resilience (Aldwin et al., 2012 [78])	Resilience is an ecological process that depends on the interplay of resources across multiple levels.	Individual: Human capital, health, finances Contextual: Social and built environment Sociocultural: Social policies, institutions	Frames resilience as being dependent on a multilevel inventory of available resources.
The Society-to-cells Model of Resilience (Szanton et al., 2010 [31])	Resilience is a dynamic, multilevel process with continuous, bidirectional influences from the macro-societal level down to the micro-cellular level.	Society Community Family Individual Physiology Cellular	Provides a comprehensive, integrated framework that links social determinants of health to biological outcomes.
An Ecological Model of Resilience (Bennett et al., 2016; Windle & Bennett, 2012 [19,79])	Resilience is shaped by the interaction of resources across three core ecological domains.	Society: Policies, Economy, services Community: Social support, cohesion, housing Individual: Psychological, biological, material resources	Provides a focused, three-tiered ecological framework commonly used in European gerontology research.
A Unified Model of Resilience and Aging (UMRA) (Wister et al., 2018 [80])	Resilience is an integrated, life-course process in which individual and socio-ecological resources are mobilized to navigate adversity, leading to different recovery trajectories.	Core Process: Adversity → Resource Activation → Protective Processes → Reintegration Resilience Domains: Social, Cognitive, Information, Physical System Functions: Plan, Absorb, Recover, Adapt	Provides a system-level model that connects individual psychological processes with disaster response functions and a life course perspective.
A Life Course Model of Multimorbidity Resilience (Wister et al., 2016 [81])	Resilience is a dynamic process of positive adaptation to the disruptions caused by multiple chronic illnesses, shaped by resources accumulated throughout the life course.	Core Process: Adversity (Multimorbidity) → Disruption → Resource Activation → Coping → Reintegration Resource Domains: Individual, Social, Environmental	Specifically frames resilience within the critical geriatric context of multimorbidity and an individual’s life course.
An Integrative, Multi-System Model of Resilience (MSMR) (Liu et al., 2017 [82])	Resilience is a multi-system construct composed of three interacting layers: a stable biological core, acquired interpersonal skills, and the external socio-ecological context.	Core (Intra-individual): Physiology, health behaviors, epigenetics Internal (Interpersonal): Psychosocial skills (coping, grit), self-regulation External (Socio-ecological): Social support, access to services, SES	Provides a tiered framework that distinguishes between stable, “trait-like” biological foundations and more malleable psychological and social factors. Advocates for including physiological markers.
System Models for Resilience in Gerontology (Klasa et al., 2021 [83])	Adapts systems engineering and disaster resilience frameworks to aging, conceptualizing resilience as a quantifiable property of an older adult nested within a multilevel socio-ecological system.	Socio-ecological spheres of influence: Individual healthy behaviors Individual determinants (e.g., genetics) Social determinants (e.g., social cohesion) Human-moderated environment Natural environment	Applies a complex systems perspective from disaster science to aging, with a strong focus on quantifying resilience to inform policy and interventions more effectively.
Resilience Model (Santos Lima et al., 2023 [84])	Resilience is a process whereby an individual leverages available resources to produce positive, adaptive behaviors.	Resources Available: Sociodemographic, experiences, social context, intrinsic aspects, health Positive Behaviors: Coping, positive perspective, emotional control	Presents resilience as a clear input-output process, distinguishing between the resources one has and the adaptive actions one takes.
Resilience Matrix (Cárdenas et al., 2010 [85])	Based on Bronfenbrenner’s theory, resilience emerges from the interaction of various processes throughout an individual’s life.	Structural processes Cultural processes Relational processes Coping processes Individual processes	Provides a process-centric view, emphasizing how diverse types of interactions contribute to resilience, rather than merely listing static factors [86,87,88,89].

**Table 4:** Characteristics of Key Resilience Measurement Tools.

Feature	Connor-Davidson Resilience Scale (CD-RISC)	Brief Resilience Scale (BRS)	Wagnild & Young Resilience Scale (RS)
Core Concept Measured	A person’s capacity to cope with and bounce back from adversity.	A person’s ability to recover from stress.	The internal strengths and positive traits that enable positive adaptation.
Theoretical Basis	Trait-based: Measures enduring personal characteristics.	Outcome-based: Measures the process of “bouncing back.”	Trait-based: Measures existential strengths.
Typical Target Population	Universally used in both community and clinical populations, including PTSD and anxiety research.	Designed for the general population; ideal for tracking changes in recovery ability over time.	Originally developed with older women, focuses on positive adaptation and is widely used in aging research.
Key Components	Personal competence, control, secure relationships, and spiritual influence.	Directly measures the ability to recover from adversity.	Purpose, perseverance, equanimity, self-reliance, existential aloneness
Key Strength	Very well-validated, with multiple versions (25, 10, and 2-item) for different settings.	Short, easy to administer, and directly measures the concept of “bouncing back.”	Focuses on positive, existential strengths, such as purpose and perseverance, rather than stress recovery.
Limitation	The 25-item version may be too lengthy for some clinical settings.	Does not measure the underlying traits that contribute to resilience.	Can be less sensitive to changes after short-term interventions compared to the BRS.
Original Reliability (Cronbach’s α)	Excellent (α = 0.89)	Excellent (α = 0.80–0.91)	Excellent (α = 0.91)
Evidence of Validity	Strong: Demonstrated good convergent validity (correlated positively with hardiness, social support; correlated negatively with perceived stress, disability) and good discriminant validity (not correlated with sexual functioning).	Good: Supported by a clear one-factor structure. Demonstrated strong convergent validity (correlated positively with optimism, purpose, social support, active coping; correlated negatively with pessimism, alexithymia, negative coping styles, stress, anxiety, depression, physical symptoms). Test–retest reliability was acceptable.	Strong: Demonstrated good concurrent validity (correlated positively with life satisfaction, morale, self-reported health; correlated negatively with depression).
Original Validation Paper	Connor & Davidson (2003) [33]	Smith et al. (2008) [34]	Wagnild & Young (1993) [35]

## Data Availability

All data generated or analyzed during this study are included in this published article and its Supplementary Information Files. The full search strings for all databases are available in the Appendix A. Further inquiries can be directed to the corresponding author.

## Supplementary Materials

The following supporting information can be downloaded at: https://www.mdpi.com/article/10.3390/geriatrics10060154/s1, the following supporting information are presented in the Supplementary File, Table S1: Summary of risk of bias assessment using a JBI checklist; Table S2: PRISMA checklist.

## Abbreviations

The following abbreviations are used in this manuscript:

WHO	World Health Organization
CD-RISC	Connor-Davidson Resilience Scale
BRS	Brief Resilience Scale
RS	Wagnild Resilience Scale
DNA	Deoxyribonucleic acid
RNA	Ribonucleic acid
PRIFOR	Physical Resilience Instrument for Older Adults
SRS	Simplified Resilience Score
SRQS	Stress Resilience Quotient Scale
RSOA	Resilience scale for older adults
ARM	Adult Resilience Measure
PRAPDI	Psychological Resilience Against Physical Difficulties Index
EORTCQLQ-C30	European Organization for Research and Treatment of Cancer Quality of Life Questionnaire

## Appendix A

Search string for PubMed is given below:

(((((((((“physical resilience”[Title]) OR (“financial resilience”[Title])) OR (“health resilience”[Title])) OR (“social resilience”[Title])) OR (“psychological resilience”[Title])) OR (“economic resilience”[Title])) OR (“spiritual resilience”[Title])) OR (“cultural resilience”[Title])) OR (“community resilience”[Title])) AND ((((((((“older adults”[Title]) OR (“aging”[Title])) OR (“elderly”[Title])) OR (“aged”[Title])) OR (“aging”[Title/Abstract])) OR (“elderly”[Title/Abstract])) OR (“older adult”[Title/Abstract])) OR (“aged”[Title/Abstract])) AND (“1 January 2017”[Date-Publication]: “30 May 2025”[Date-Publication])

Search string for the PsycNet is given below:

Title: physical resilience OR Title: financial resilience OR Title: health resilience OR Title: social resilience OR Title: psychological resilience OR Title: economic resilience OR Title: spiritual resilience OR Title: cultural resilience OR Title: community resilience AND Title: older adults OR Title: aging OR Title: elderly OR Title: aged OR Abstract: aging OR Abstract: elderly OR Abstract: older adult OR Abstract: aged AND Age Group: Aged (65 yrs & older) AND Year: 2017 To 2025

Search string for the JSTOR is given below:

ti:(resilience) AND ti:(“older adults” OR elderly OR aging OR aged)
