# Supplementary material for: A Multidimensional Perspective on Resilience in Later Life: A Systematic Literature Review of Protective Factors and Adaptive Processes in Ageing
Source: Geriatrics (Basel). 2025 Nov 21;10(6):154. doi: 10.3390/geriatrics10060154 (PMC12641927; doi:10.3390/geriatrics10060154)
Supplement: Supplementary file 1 [file geriatrics-10-00154-s001.zip › geriatrics-3856339-supplementary.pdf]

Supplementary Materials

Table S1: Summary of risk of bias assessment using a JBI checklist

Study	Q1	Q2	Q3	Q4	Q5	Q6	Q7	Q8	Q9	Q10	Q11	Overall
**Cross-sectional study**												
Siu Yu Zoe Lau et al., 2018	Y	Y	U	Y	NA	NA	Y	Y	-	-	-	MODERATE
Jyväkorpi et al., 2018	Y	Y	U	Y	U	U	Y	Y	-	-	-	MODERATE
Olson et al., 2021	Y	Y	U	Y	U	U	Y	Y	-	-	-	MODERATE
Filiz Kunuroglu et al., 2021	Y	Y	Y	Y	NA	NA	Y		-	-	-	MODERATE
Sarah E. Remm et al., 2023	Y	Y	Y	Y	NA	NA	Y	Y	-	-	-	HIGH
Costenoble et al., 2021	Y	Y	Y	U	U	U	Y	Y	-	-	-	MODERATE
Yan Wang et al., 2024	Y	Y	Y	Y	Y	U	Y	Y	-	-	-	HIGH
Andrew V Wister et al., 2024	Y	Y	U	U	NA	NA	Y	Y	-	-	-	MODERATE
Mitra ZAFARI et al., 2023	Y	Y	Y	Y	NA	NA	U	Y	-	-	-	MODERATE
Jung-Ran Kim et al., 2023	Y	Y	U	U	NA	NA	Y	Y	-	-	-	MODERATE
Rodrigo C.M. Silva et al., 2022	Y	Y	U	Y	U	U	Y	Y	-	-	-	MODERATE
Marcia Morete et al., 2018	Y	Y	Y	Y	NA	NA	Y	Y	-	-	-	HIGH
Faezh Kondabi et al., 2017	Y	Y	Y	U	U	U	Y	Y	-	-	-	MODERATE
R. H. Asch et al., 2021	Y	Y	Y	Y	Y	U	Y	Y	-	-	-	HIGH
Yang-Tzu Li et al., 2022	Y	Y	Y	U	NA	NA	Y	Y	-	-	-	MODERATE
Laird KT et al., 2019	Y	Y	Y	Y	U	U	Y	Y				HIGH
**Cohort study**												
Kim et al., 2024b	Y	Y	Y	NA	NA	U	Y	U	NA	NA	Y	MODERATE
Gijzel et al., 2019	Y	Y	Y	NA	NA	U	Y	Y	Y	Y	Y	MODERATE
Adele M. Taylor et al., 2019	Y	Y	Y	NA	NA	Y	Y	Y	Y	Y	Y	HIGH
Rodrigues and Tavares, 2024b	U	Y	Y	NA	NA	NA	Y	Y	Y	Y	Y	MODERATE

Stenroth et al., 2023	Y	Y	Y	NA	NA	NA	Y	Y	Y	Y	Y	HIGH
Milou J. Angevaare et al., 2023	U	U	Y	Y	Y	Y	Y	NA	NA	NA	Y	MODERATE
Miller et al., 2024	U	Y	Y	NA	NA	NA	Y	U	U	U	Y	MODERATE
Rolandi et al., 2024	U	Y	Y	NA	NA	NA	Y	Y	Y	Y	Y	MODERATE
Hao et al., 2023	Y	Y	Y	NA	NA	NA	Y	Y	Y	Y	Y	HIGH
Guo-qing Jiang et al., 2024	Y	Y	Y	U	U	Y	Y	U	U	U	Y	MODERATE
Susan P. Phillips et al., 2016	Y	Y	Y	U	U	Y	Y	U	U	U	Y	MODERATE
Drazich et al., 2024	Y	Y	Y	U	U	U	Y	U	U	U	Y	MODERATE
Gijzel et al., 2017	Y	Y	Y	NA	NA	U	Y	Y	Y	Y	Y	MODERATE
Hu et al., 2021	Y	Y	Y	NA	NA	U	Y	Y	Y	Y	Y	MODERATE
Hu et al., 2023	Y	Y	Y	U	U	U	Y	Y	Y	Y	Y	MODERATE
Sugawara et al., 2022	U	Y	Y	U	U	U	Y	U	U	U	Y	MODERATE
Taylor and Carr, 2020	Y	Y	Y	NA	NA	U	Y	Y	Y	Y	Y	MODERATE
Lenti et al., 2022	Y	Y	Y	Y	Y	U	Y	U	Y	Y	Y	MODERATE
Rebagliati et al., 2017	Y	Y	Y	U	U	Y	Y	U	U	U	Y	MODERATE
Choi and Kim, 2024	Y	Y	Y	U	U	Y	Y	U	U	U	Y	MODERATE
Kim et al., 2024a	Y	Y	Y	U	U	U	Y	U	U	U	Y	MODERATE
Costenoble et al., 2023	U	Y	U	NA	NA	U	Y	Y	Y	Y	Y	MODERATE
Ye et al., 2024	Y	Y	Y	NA	NA	U	Y	Y	Y	Y	Y	HIGH
Yang and Wen, 2017	Y	Y	Y	NA	NA	U	Y	U	U	U	Y	MODERATE
Akkila et al., 2023	Y	Y	Y	NA	NA	U	Y	U	U	U	Y	MODERATE
Kolk et al., 2021	Y	Y	Y	U	U	U	Y	U	U	U	Y	MODERATE
Resnick B et al., 2019	Y	Y	Y	U	U	Y	Y	Y	Y	Y	Y	HIGH
**Quasi-experimental study**												
Jonathan D. Bartholomaeus et al., 2019	Y	U	U	U	Y	Y	Y	Y	Y	-	-	MODERATE

Questions

Cross-sectional study

Q1: Were the criteria for inclusion in the sample clearly defined?

Q2: Were the study subjects and the setting described in detail?

Q3: Was the exposure measured in a valid and reliable way?

Q4: Were objective, standard criteria used for measurement of the condition?

Q5: Were confounding factors identified?

Q6: Were strategies to deal with confounding factors stated?

Q7: Were the outcomes measured in a valid and reliable way?

Q8: Was appropriate statistical analysis used?

Cohort study

Q1: Were the two groups similar and recruited from the same population?

Q2: Were the exposures measured similarly to assign people to both exposed and unexposed groups?

Q3: Was the exposure measured in a valid and reliable way?

Q4: Were confounding factors identified?

Q5: Were strategies to deal with confounding factors stated?

Q6: Were the groups/participants free of the outcome at the start of the study (or at the moment of exposure)?

Q7: Were the outcomes measured in a valid and reliable way?

Q8: Was the follow up time reported and sufficient to be long enough for outcomes to occur?

Q9: Was follow up complete, and if not, were the reasons to loss to follow up described and explored?

Q10: Were strategies to address incomplete follow up utilized?

Q11: Was appropriate statistical analysis used?

Quasi-experimental study

Q1: Is it clear in the study what is the "cause" and what is the "effect" (i.e. there is no confusion about which variable comes first)?

Q2: Was there a control group?

Q3: Were participants included in any comparisons similar?

Q4: Were the participants included in any comparisons receiving similar treatment/care, other than the exposure or intervention of interest?

Q5: Were there multiple measurements of the outcome, both pre and post the intervention/exposure?

Q6: Were the outcomes of participants included in any comparisons measured in the same way?

Q7: Were outcomes measured in a reliable way?

Q8: Was follow-up complete and if not, were differences between groups in terms of their follow-up adequately described and analyzed?

Q9: Was appropriate statistical analysis used?

Table S2: PRISMA checklist

Section and Topic	Item #	Checklist item	Location where item is reported
**TITLE**			

Section and Topic	Item #	Checklist item	Location where item is reported
Title	1	Identify the report as a systematic review.	P: 1
**ABSTRACT**			
Abstract	2	See the PRISMA 2020 for Abstracts checklist.	P: 1
**INTRODUCTION**			
Rationale	3	Describe the rationale for the review in the context of existing knowledge.	P: 1
Objectives	4	Provide an explicit statement of the objective(s) or question(s) the review addresses.	P: 3
**METHODS**			
Eligibility criteria	5	Specify the inclusion and exclusion criteria for the review and how studies were grouped for the syntheses.	P:4
Information sources	6	Specify all databases, registers, websites, organisations, reference lists and other sources searched or consulted to identify studies. Specify the date when each source was last searched or consulted.	P:3
Search strategy	7	Present the full search strategies for all databases, registers and websites, including any filters and limits used.	P:3
Selection process	8	Specify the methods used to decide whether a study met the inclusion criteria of the review, including how many reviewers screened each record and each report retrieved, whether they worked	P: 4

Section and Topic	Item #	Checklist item	Location where item is reported
		independently, and if applicable, details of automation tools used in the process.	
Data collection process	9	Specify the methods used to collect data from reports, including how many reviewers collected data from each report, whether they worked independently, any processes for obtaining or confirming data from study investigators, and if applicable, details of automation tools used in the process.	P:4
Data items	10a	List and define all outcomes for which data were sought. Specify whether all results that were compatible with each outcome domain in each study were sought (e.g. for all measures, time points, analyses), and if not, the methods used to decide which results to collect.	P:3
	10b	List and define all other variables for which data were sought (e.g. participant and intervention characteristics, funding sources). Describe any assumptions made about any missing or unclear information.	P:3
Study risk of bias assessment	11	Specify the methods used to assess risk of bias in the included studies, including details of the tool(s) used, how many reviewers assessed each study and whether they worked independently, and if applicable, details of automation tools used in the process.	P:4

Section and Topic	Item #	Checklist item	Location where item is reported
Effect measures	12	Specify for each outcome the effect measure(s) (e.g. risk ratio, mean difference) used in the synthesis or presentation of results.	Not Reported
Synthesis methods	13a	Describe the processes used to decide which studies were eligible for each synthesis (e.g. tabulating the study intervention characteristics and comparing against the planned groups for each synthesis (item #5)).	P: 5
	13b	Describe any methods required to prepare the data for presentation or synthesis, such as handling of missing summary statistics, or data conversions.	Not Reported
	13c	Describe any methods used to tabulate or visually display results of individual studies and syntheses.	P: 4
	13d	Describe any methods used to synthesize results and provide a rationale for the choice(s). If meta-analysis was performed, describe the model(s), method(s) to identify the presence and extent of statistical heterogeneity, and software package(s) used.	Not Reported
	13e	Describe any methods used to explore possible causes of heterogeneity among study results (e.g. subgroup analysis, meta-regression).	Not Reported
	13f	Describe any sensitivity analyses conducted to assess robustness of the synthesized results.	P:4
Reporting bias assessment	14	Describe any methods used to assess risk of bias due to missing results in a synthesis (arising from reporting biases).	Not Reported

Section and Topic	Item #	Checklist item	Location where item is reported
Certainty assessment	15	Describe any methods used to assess certainty (or confidence) in the body of evidence for an outcome.	Not Reported
**RESULTS**			
Study selection	16a	Describe the results of the search and selection process, from the number of records identified in the search to the number of studies included in the review, ideally using a flow diagram.	P: 6
	16b	Cite studies that might appear to meet the inclusion criteria, but which were excluded, and explain why they were excluded.	Figure 1 (lists of full citation is not added)
Study characteristics	17	Cite each included study and present its characteristics.	P: 7
Risk of bias in studies	18	Present assessments of risk of bias for each included study.	P: 8
Results of individual studies	19	For all outcomes, present, for each study: (a) summary statistics for each group (where appropriate) and (b) an effect estimate and its precision (e.g. confidence/credible interval), ideally using structured tables or plots.	P: 7
Results of syntheses	20a	For each synthesis, briefly summarise the characteristics and risk of bias among contributing studies.	P: 10
	20b	Present results of all statistical syntheses conducted. If meta-analysis was done, present for each the summary estimate and its precision (e.g. confidence/credible interval) and measures of	P: 10

Section and Topic	Item #	Checklist item	Location where item is reported
		statistical heterogeneity. If comparing groups, describe the direction of the effect.	
	20c	Present results of all investigations of possible causes of heterogeneity among study results.	Not Reported
	20d	Present results of all sensitivity analyses conducted to assess the robustness of the synthesized results.	P: 7
Reporting biases	21	Present assessments of risk of bias due to missing results (arising from reporting biases) for each synthesis assessed.	Not Reported
Certainty of evidence	22	Present assessments of certainty (or confidence) in the body of evidence for each outcome assessed.	Not Reported
**DISCUSSION**			
Discussion	23a	Provide a general interpretation of the results in the context of other evidence.	P: 16
	23b	Discuss any limitations of the evidence included in the review.	P: 19
	23c	Discuss any limitations of the review processes used.	P: 19
	23d	Discuss implications of the results for practice, policy, and future research.	P: 17
**OTHER INFORMATION**			
Registration and protocol	24a	Provide registration information for the review, including register name and registration number, or state that the review was not registered.	P: 20

Section and Topic	Item #	Checklist item	Location where item is reported
	24b	Indicate where the review protocol can be accessed, or state that a protocol was not prepared.	P: 20
	24c	Describe and explain any amendments to information provided at registration or in the protocol.	Not Reported
Support	25	Describe sources of financial or non-financial support for the review, and the role of the funders or sponsors in the review.	P: 20
Competing interests	26	Declare any competing interests of review authors.	P: 21
Availability of data, code and other materials	27	Report which of the following are publicly available and where they can be found: template data collection forms; data extracted from included studies; data used for all analyses; analytic code; any other materials used in the review.	P: 21
